# Sex differences in self-regulation in early, middle and late adolescence: A large-scale cross-sectional study

**DOI:** 10.1371/journal.pone.0227607

**Published:** 2020-01-13

**Authors:** M. A. J. van Tetering, A. M. van der Laan, C. H. de Kogel, R. H. M. de Groot, J. Jolles

**Affiliations:** 1 Centre for Brain & Learning, Faculty of Behavioural and Movement Sciences, Vrije Universiteit Amsterdam, Amsterdam, The Netherlands; 2 Research and Documentation Centre (WODC), Ministry of Justice and Security, The Hague, The Netherlands; 3 Institute, Research Centre for Learning, Teaching, and Technology, Open University of the Netherlands, Heerlen, The Netherlands; 4 NUTRIM School of Nutrition and Translational Research in Metabolism, Maastricht University, Maastricht, The Netherlands; Institute of Physiology and Basic Medicine, RUSSIAN FEDERATION

## Abstract

This large-scale cross-sectional study had the aim to investigate whether adolescent males and females differ in self-perceived self-regulation. The large sample size allowed us to investigate sex differences in three age-groups of young (n = 161), middle (n = 133) and late (n = 159) adolescents. Self-regulation was evaluated with a self-report questionnaire, the Amsterdam Executive Functioning Inventory (AEFI). This questionnaire gives a proxi for three executive functions that are important for proper self-regulation: (1) self-control & self-monitoring, (2) attention, and (3) planning & initiative taking. Results revealed clear sex differences in the self-regulation as perceived by mid-adolescents (i.e., 13–16 years). In this age period, females evaluated their attention higher than males, and they reported higher levels of self-control & self-monitoring. Our findings offer important new insights with respect to the decision making, academic achievements and behaviour of 13-16-year olds. Self-regulation is known to have a central role in academic achievement and in behavioural organisation. The sex differences in self-regulation in mid-adolescence may therefore explain part of the difference which males and females in this age-group exhibit in academic achievements and behavioural organisations. The results imply that self-regulation may be a relevant intervention target: rather than focussing on changing behaviour, interventions may focus more on self-insights and thereby changing the adolescent’s *perceptions about their behaviour*. Increased self-insight may have the potency to actually change behaviour, which might be an interesting target for future investigation.

## Introduction

Self-regulation is a neuropsychological skill, which continues to develop from infancy through childhood into late adolescence. During this period, adolescents become better able to control their impulses ([1]). They also increasingly master the ability to pay attention in more complex situations and to focus on tasks for longer periods of time ([1], [2]). In addition, they acquire the skills to plan future behaviour on the short and longer term, and to monitor and control thoughts, emotions and behaviour ([1], [2], [3], [4]). Although all individuals grow in their self-regulation over the long period of adolescence ([1], [2], [4], [5], [6], [7]), there are substantial individual differences in the pace at which this skill improves. Adolescents with poor self-regulation encounter more difficulties in concentrating at school and organising homework, and in developing stable and healthy friendships. They also show more behavioural problems than adolescents with higher levels of self-regulation (e.g., [5], [6], [7], [8], [9], [10]). Conversely, youth with higher levels of self-regulation appear to be better resistant to negative temptations and peer pressure (e.g., [5], [6], [8], [9], [11]). They are also better able to organise their learning assignments for school ([7]). Skills in self-regulation thus impact behaviour and school achievement, and good self-regulation aids in resisting the temptations that the peer group has to offer. Given the important differences which exist between adolescents in school performance and learning motivation, and in actions that are the manifestation of poor self-regulation, it is of importance to evaluate self-regulation in various groups of adolescents. The present large-scale study was set up to investigate the notion that the sex of the adolescent is an important factor in determining individual differences in self-perceived self-regulation.

Sex differences in school performance and in many aspects of the cognition and behaviour of adolescents have been well-established, and are reported in many industrialised countries (e.g., [12], [13], [14], [15], [16], [17]). With respect to school performance, adolescent males more often flow to lower educational tracks than adolescent females, and their school drop-out rates are higher ([17]). The notion taken in the present paper is that sex differences in the pace at which self-regulation develops over the course of adolescence may contribute to these male-female differences in school performance. This is substantiated by the results of our earlier studies which have reported on the importance of self-regulation to academic achievement (e.g., [7], [18], [19]). For example, Baars et al. [18], investigated the relation between self-regulation and academic achievement in 17-to 21-year olds. Results showed that students with better self-regulation at the start of the first year of their study obtained more study-credits than students with lower self-regulation at the end of that year. Based upon our earlier research (see also [19] and [20]), we hypothesise that self-regulation develops at a slower pace in adolescent males than in females. A slower development of self-regulation skills in adolescent males would explain why they experience more problems with planning homework, learning for a test, or concentrating at school than females (e.g., [21], [22]). This could account for their worse performance at school as compared to females. Support for this hypothesis comes from the findings described in our earlier cross-sectional study ([22]). In this study we found that teachers observed more problems with the organisation of schoolwork and with impulse control in males than in females at the age of 8–12 years.

Additional support for the notion that adolescent males and females differ in self-regulation during the period of adolescence comes from well-established sex differences in the occurrence of behavioural problems. Adolescent males appear to outnumber adolescent females in fatal accidents ([23]), gambling ([24]) and crime ([25], [26], [27], [28]). Evidence accumulates that lower levels of self-regulation are a risk factor for the development of these psycho-social-behavioural problems. For instance, Shulman and colleagues [28] reported on sex differences in the developmental trajectories of impulse control from early adolescence to adulthood. Their study revealed that the ability to control impulses improved more gradually in males than in females. These researchers therefore suggest that the window of heightened vulnerability to risk-taking during adolescence may be more protracted for adolescent males than for females ([28]). Sex differences in (the development of) self-regulation could thus be an important factor in determining differences in cognition, academic performances and the behaviour of adolescent males and females.

Circumstantial evidence for the existence of possible sex differences in the development of self-regulation is provided by neuroimaging studies. For instance, Lenroot and Giedd [29] showed that adolescent females and males exhibit a four years difference in the age at which their brains reach the greatest volume: average age is 10.5 years for females and 14.5 years for males. These authors thus show that the brain maturation of males is lagging behind that of females in the periods of early and middle adolescence. Their finding has been confirmed by other researchers (e.g., [30], [31]). This difference suggests that sex could be regarded as possible factor contributing to individual differences in the cognitive development during adolescence, which is dependent upon brain maturation. It is therefore likely that adolescent males and females differ in the way they regulate behaviour during adolescence and that a maturational lag in the development of self-regulation in adolescent males is an important factor in this respect. To investigate this was the aim of the investigation described in the present paper.

In the present study, adolescents were asked to evaluate their levels of self-regulation on a self-report questionnaire, and it was monitored whether adolescent males and females differ in their evaluations. The use of self-reports is the method of choice when information is needed on the self-insights and perceptions of individuals. Consequently, self-report questionnaires are used in studies on anxiety, depression and other aspects of mental health, and in studies of memory complaints and executive functions (e.g., [32], [33], [34], [35], [36], [37], [38], [39], [40]). In the present study, we evaluate the self-reports of adolescents using the Amsterdam Executive Function Inventory. This measure has been used in several large-scale investigations in adolescents (e.g., [7], [18], [22], [41]). The AEFI is a measure which is similar to the widely used BRIEF (Behavior Rating Inventory of Executive Function; [33], [37], [42]). A difference in advantage to the AEFI is its length, which makes it easier to use in large-scale studies: the AEFI uses 13 items whereas the BRIEF uses 68 items. Another advantage to the AEFI is that it focuses primarily on self-insight and self-regulation, whereas the BRIEF evaluates various different executive functions such as emotion regulation and others, which were not relevant for our research questions. Examples of two major items of the AEFI are ‘I am well-organised. For example, I am good at planning what I need to do during a day” and “It takes a lot of time for me to finish tasks” ([22]). Other studies on sex differences in self-regulation primarily focused on emotional regulation ([43]), or have used observer reports ([44]) or behavioural tasks to measure self-regulation (see the meta-analysis of [45]). Previous studies have also focused on other cognitive domains, such as impulse control ([46]) in relation to sensation seeking or on emotional control (e.g., [47], [48]). Impulse control is an important aspect of executive functioning (see [2] and [49]), but it is a distinct skill from self-evaluation and self-regulation, which is the focus of our study and its predecessors ([7], [18], [22], [41]).

Regarding the setup and design of our study, the factor ‘age’ deserves some further elaboration. As noted, the goal of this large-scale cross-sectional study was to investigate sex differences in self-regulation in adolescence by self-report. In such a study, it is imperative to control for the factor age. This is necessary in view of the substantial development which takes place over the course of adolescence. The period of adolescence (starts around 10 years of age through at least 22 years, e.g. [50]) is characterised by three or more phases; young, middle and late adolescence. Curtis [51] elaborates on this point and describes quite some differences between these phases in both psychological and physical development, and also in social behaviour and cognition. It is therefore possible that difference between adolescent males and females in self-regulation can be different in the phases discerned by Steinberg & Morris [50] and by Curtis [51]. Therefore, the present study evaluates the possible impact of sex on self-regulation in the three separate age group that were indicated by Steinberg & Morris [50] and Curtis [51].

A final note about the composition of our sample is that we have designed our study to focus on adolescents who live in and have grown up in a Western country. This was done in order to exclude heterogeneity due to age-extrinsic factors, notably cultural background and ethnicity (see for an elaboration on the importance: [16], [45], [52]: their papers describe substantial confounding by these factors). Note that adolescents who have another cultural heritage, language or socio-economic background (e.g. children of immigrants) can show substantial differences in neuropsychological development (e.g., [16], [45], [52]). Our study sample was therefore well-controlled with respect to the background of the subjects, and the study sample can be regarded as relatively homogeneous with respect to ethnic and cultural background.

In short, this paper describes a large cross-sectional study involving 453 adolescents in which adolescent males and females are compared with regard to their self-regulation using self-reports. The study was part of the repeated cross-sectional WODC Youth Delinquency Survey wave which included 5, 266 adolescents aged 10–23 years old ([53]). Our hypothesis was that males are worse in self-regulation than females. More specifically, male-female differences in academic achievement and behaviour have especially been reported in the periods of early and middle adolescence. These differences are less frequently reported in the period of late adolescence ([54], [55]). We therefore expected that sex differences in self-regulation would especially be found in early and middle adolescence.

There is applied relevance of the study in view of the fact that self-regulation has a central role in academic achievement and behavioural problems. Thus, any evidence for sex differences in particular periods of adolescence may offer important new insights into the underpinnings of adolescents’ daily life decisions, academic achievements and behaviour. It may also offer new insights with respect to applied interventions aimed at improving self-regulation skills. The focus on self-reports may offer applied implications: rather than focussing on changing behaviour, interventions may focus more on self-perceptions and thereby change the adolescent’s *perceptions about their behaviour* and the control which adolescents experience over their behaviour.

## Methods

### Procedure

Part of the dataset from the WODC Youth Delinquency Survey wave 2015 ([53]) was used for the present study. The 2015 wave of this repeated cross-sectional survey was carried out in the Netherlands between January and June 2015. A stratified random sampling method was used in which the strata were based on age (10–23 years old) and nationality (including Moroccan, Turkish, Surinamese and Antillean or Aruban). Within these strata, a random selection was made from the home addresses of adolescents from the Municipal Base Registry (MBR). This register contains all legally registered inhabitants in the Netherlands. A total of 5, 266 individuals aged 10 to 23 were selected from the MBR. These individuals can be considered as the basis population out of which the study sample for the present study has been drawn. These individuals received an information letter and gave oral consent. If individuals were aged 15 years or younger, consent was given by caregivers. The interview contained a broad range of questions related to demographics (i.e., characteristics of the family) and risk factors for delinquency (e.g., regarding parenting styles, peer delinquency). These questions were administered by means of computer assisted personal interviewing (CAPI). The interview also contained items regarding self-regulation (e.g., [22], [41], [56]). These items were administered by computer assisted self-interviewing (CASI). Basic demographic variables (e.g. sex, ethnicity, age and social economic status of the family) were extracted from the System of Social Statistical Datasets of Statistic Netherlands ([57]), and connected at the micro-level to the database. The research was approved by the Central Bureau of Statistics (CBS), the Netherlands.

### Participants

A total of 3, 188 individuals aged between 10–23 years agreed to be interviewed for the WODC Youth Delinquency Survey wave 2015. The response rate was 60,5%, which is acceptable ([58]). Of these individuals, only participants aged between 10–19 years were selected for the present study. In addition, we have asked participants to indicate their own heritage. Only those participants who indicated to have a Dutch heritage (*n* = 593) were included into our study sample. These selection criteria followed from the central research question of this study, which targeted adolescents who had a life and learning history that is representative for individuals growing up in Western countries. This was done in order to control for cultural differences between adolescents that could influence our main outcome measure of interest, as it has extensively been reported that cultural background influences neuropsychological development (see for instance [16], [45], [52]). In order to control for the confounder ‘cultural background’, we thus chose to focus on a selective group of adolescents with a similar heritage.

Additionally, excluded from participation were those participants who were characterised by missing data on the items related to self-regulation (*n* = 2) or if they had repeated a grade (*n* = 138). The decision to exclude individuals who repeated a grade was based upon the consideration that the cognitive development of these individuals could lag behind that of others of the same age who have a regular academic performance (see also [7]). This notion was confirmed by our post-hoc one-way analyses of variances (ANOVAs) showing that self-regulation was lower for individuals who repeated a grade compared to those who did not repeat a grade. This was found on the total score for self-regulation (*F* (1, 589) = 11.54, *p* < .01), as well as on two of the three subscales underlying this total score for self-regulation, i.e., self-control & self-monitoring (*F* (1, 589) = 3.89, *p* < .05) and attention (*F* (1, 589) = 20.26, *p* < .01). By excluding adolescents who repeated a grade, we thus additionally controlled for the confounder ‘age’ by homogenising the study sample with respect to individual differences in cognition within grades.

The remaining study sample consisted of *n* = 453 Dutch participants. Their average age was 14.1 years (*SE* = 0.13). Of these adolescents, 53.2% were female. ANOVAs revealed that the mean age of adolescent males (*M* = 13.79, *SE* = 0.19) and that of females (*M* = 14.27, *SE* = 0.18) in the total study population was the same, *F* (1, 451) = 3.34, *p* = .07, *η*_*p*_ < 0.01.

The participants were divided into three age groups: one group with a mean age of 10.9 years (*SE* = 0.06) (*n* = 161; age range = 10.00–12.99 years; 48.5% female), a second group with a mean age of 14.0 years (*SE* = 0.07) (*n* = 133; age range 13.00–15.99 years; 52.6% female), and a third group with a mean age of 17.3 (*SE* = 0.07) (*n* = 159; age range = 16.00–18.99 years; 58.5% female). Again, ANOVAs revealed that the average age of adolescent females and males did not significantly differ in the first (*F* (1,159) = 1.49; *p* = .22, *η*_*p*_ *<* 0.01), second (*F* (1, 131) = 1.40, *p* = .24, *η*_*p*_ = 0.01) and third age group (*F* (1, 157) = 0.45, *p* = .50, *η*_*p*_ < 0.01). Table 1 gives an overview of the number of participants and the mean age for adolescent males and females per age group.

**Table 1:** Number of participants and mean age for adolescent males and females per age group.

			Age groups			
	1		2		3	
	10–12 years		13–15 years		16–19 years	
	Males	Females	Males	Females	Males	Females
**N**	83	78	63	70	66	93
**Age (M) SE**	11.0 (0.09)	10.9 (0.09)	13.9 (0.10)	14.06 (0.10)	17.20 (0.12)	17.29 (0.09)

### Dependent measure: The Amsterdam Executive Functioning Inventory (AEFI)

The Amsterdam Executive Functioning Inventory (AEFI) is a self-report questionnaire which has been developed to measure a proxy of self-regulation ([41]). It has been used in earlier studies to gain insight into the evaluations of individuals about their behaviour ([7], [18], [22], [59]). The AEFI consists of 13 items. The total score of these items represents a robust proxy of self-regulation. It is a composite score of the three subscales which represent three dimensions of executive functioning, that is, (1) *self-control & self-monitoring* (e.g., “I often react too fast, I have done or said something before it is my turn”), (2) *attention* (e.g., “I am not able to focus on the same topic for a long period of time”), and (3) *planning & initiative taking* (e.g., “I am good at planning what I need to do during a day”). The items that belong to these dimensions were a priori clustered together.

Earlier studies reported that the internal consistency of these subscales was sufficient (e.g., [7], [22], [41]). For this study, the reliability and internal consistency of the AEFI were examined again to ensure that both were acceptable in the present study population. Results revealed that the Cronbach’s alphas (ranged between 0.4 and 0.7) were essentially the same as in previous studies. In addition, the corrected item-scale correlations (i.e., the correlations between items and scale scores that did not include the items being evaluated), were calculated. These correlations should be ≥ .30 ([60]). For shorter scales, the corrected item-scale values provide a better index of internal consistency and reliability than Cronbach's alpha, because Cronbach’s alpha values are not only a function of the height of the inter-correlations between the items of a scale, but also a function of the number of items on that scale ([61]). Again, the inter-item correlations in the present study were essentially the same as those in Van Der Elst and colleagues [41] and in van Tetering & Jolles [7], with the exception of somewhat lower correlations on the *planning & initiative taking* scale in the late adolescent group. Yet, together with the Cronbach alpha values, we conclude that the inter-item correlations were acceptable (see S1 Table in the supporting information).

In the present study, there were minor differences in the versions of the AEFI used for 10– to 12–year-old individuals, and the one for 13– to 19–year-old individuals. These differences pertained to some examples given to explain the items to individuals aged 10– to 12–years old (in primary school). This was done to make the items age appropriate (see [7], [22], [41]). All participants were asked to indicate how well each item of the AEFI suited them by endorsing one of three responses on a 3-point Likert scale: 1 = ‘not true,’ 2 = ‘partly true,’ or 3 = ‘true’. Items 1, 4, 5, 6, 7, 8, 11, 12, and 13 were-reverse coded, and the total score of all items was calculated so that higher scores were indicative of better self-regulation.

### Statistical analyses

Normality distributions were investigated by visual inspection of the histograms and the normal probability plots. Both were adequate. Next, ANOVAs were performed with age group (age groups 1, 2 and 3) and sex (adolescent males versus females) as independent variables and the four outcome measures of the AEFI (i.e., *total AEFI score* as primary outcome measure, and *self-control & self-monitoring*, *attention*, *and planning & initiative taking* as secondary outcome measures) as dependent variables. *P*-values ≤ .05 were considered statistically significant. If the analyses revealed a significant main effect of age group on any of the outcome measures, additional one-way ANOVAs were performed to investigate the differences between the three age groups more specifically: Mean of age group 1 was compared to mean of age group 2 and mean of age group 2 was compared to mean of age group 3. Only the consecutive classes were compared to investigate whether self-evaluations changed with age. For these additional analyses, Modified Hochberg correction was used to control for multiple testing issues. As this correction is less conservative than more traditional procedures (such as the Bonferroni correction), it reduces the chance of a type II error (see [62]). According to this correction, *p*-values of ≤ .03 were considered statistically significant ([62]).

Finally, post-hoc analyses were performed to investigate sex differences in the three age groups separately. Accordingly, one-way ANOVAs were performed with sex as independent variable and the four outcome measures of the AEFI as depended variables. These analyses were performed in each age group. Again, Modified Hochberg correction was used to control for multiple testing issues. Because of the correction, *p*-values of ≤ .03 were considered statistically significant ([62]). Partial eta squared (*η*_*p*_^*2*^) was calculated as a measure of effect-size for the significant outcomes. Analyses were performed in SPSS Statistics 24.

## Results

### Sex differences on the primary outcome measure: Self-regulation

#### Self-regulation (total AEFI score)

Analyses were performed to investigate the main effects of age group and sex, and the interaction between age group and sex on *self-regulation* (i.e., the total AEFI score). Results revealed a significant interaction between age group and sex, *F* (2, 447) = 4.12, *p* = .02, *η*_*p*_^*2*^ = 0.02). Main effects of age group (*F* (2, 447) = 0.02, *p* = .98, *η*_*p*_^*2*^ = 0.00) and sex (*F* (1, 477) = 3.59, *p* = .68, *η*_*p*_^*2*^ = 0.00) were not statistically significant. Results are presented in Table 2.

**Table 2:** Main effects and interaction effects on the AEFI scales.

	*Outcomes*		
	F	*p*-values	*η*_p_^2^
*Self-regulation (total AEFI score)*			
Age group	0.02	.98	0.00
Sex	3.59	.68	0.00
Age group x Sex	4.12	.02*	0.02
*Self-control & self-monitoring*			
Age group	0.12	.89	<0.01
Sex	0.19	.66	<0.01
Age group x Sex	3.45	.03*	0.02
*Attention*			
Age group	3.70	.03*	0.02
Sex	3.10	.08	<0.01
Age group x Sex	2.10	.12	<0.01
*Planning & initiative taking*			
Age group	2.84	.06	0.01
Sex	1.12	.29	<0.01
Age group x Sex	2.15	.12	0.01

*p ≤ .03

Post-hoc analyses in which sex differences were investigated in each of the three age groups allowed a further evaluation of the significant interaction effect on *self-regulation*. The sex difference was statistically significant in the second age group, *F* (1, 132) = 7.46, *p* < .01, *η*_*p*_^*2*^ = 0.05. *Self-regulation* of adolescent females (*M* = 17.67, *SE* = 0.51) was higher than that of adolescent males (*M* = 15.76, *SE* = 0.48). The sex differences in the first (*F* (1, 159) = 0.16, *p* = .69, *η*_*p*_^*2*^ = 0.01) and third (*F* (1, 157) = 1.96, *p* = .16, *η*_*p*_^*2*^ = 0.01) age groups were not statistically significant. Accordingly, the statistically significant sex-effect found on the total group was due to a large difference between males and females in middle adolescence. Mean and standard errors for adolescent males and females in each age group are presented in Table 3.

**Table 3:** Mean and standard errors of adolescent males and females in early, middle and late adolescence on the AEFI scales.

	Age groups					
	1		2		3	
	10–12 years		13–15 years		16–19 years	
	*n* = 83	*n* = 78	*n* = 63	*n* = 70	*n* = 66	*n* = 93
	M (SE)	M (SE)	M (SE)	M (SE)	M (SE)	M (SE)
Self-regulation (total AEFI score)	16.78 (0.49)	16.49 (0.55)	15.76 (0.48)	17.67 (0.51)*	17.26 (0.55)	16.18 (0.52)
*AEFI subscales*						
Self-control & self-monitoring	6.46 (0.24)	6.53 (0.27)	6.22 (0.28)	7.01 (0.24)*	6.83 (0.24)	6.25 (0.25)
Attention	3.35 (0.19)	3.39 (0.19)	2.52 (0.21)	3.29 (0.19)*	2.89 (0.20)	2.95 (0.20)
Planning & initiative taking	6.98 (0.22)	6.58 (0.21)	7.02 (0.20)	7.37 (0.23)	7.53 (0.24)	6.99 (0.22)

*p ≤ .03

### Sex differences on the secondary outcome measures

#### Self-control & self-monitoring

Analyses were performed to investigate the main effects of age group and sex, and the interaction between age group and sex on the AEFI subscale *self-control & self-monitoring*. Results revealed a significant interaction between age group and sex, *F* (2, 447) = 3.45, *p* = .03, *η*_*p*_^*2*^ = 0.02. The main effects of age group (*F* (2, 447) = 0.12, *p* = .89, *η*_*p*_^*2*^ < 0.01) and sex (*F* (1, 447) = 0.19, *p* = .66, *η*_*p*_^*2*^ < 0.01) were not statistically significant. Results are presented in Table 2.

Post-hoc analyses in which sex differences were investigated in each of the three age groups separately allowed us to further evaluate the significant interaction effect on the subscale *self-control & self-monitoring*. Results revealed a significant sex difference in the second age group, *F* (1, 131) = 4.67, *p* = .03, *η*_*p*_^*2*^ = 0.03. Adolescent females (*M* = 7.01, *SE* = 0.24) evaluated self-control & self-monitoring higher than males (*M* = 6.22, *SE* = 0.28). The sex differences in the first (*F* (1, 159) = 0.04, *p* = .85, *η*_*p*_^*2*^ = 0.00) and third (*F* (1, 157) = 2.65, *p* = .11, *η*_*p*_^*2*^ = 0.02) age groups were not statistically significant. Mean and standard errors for adolescent males and females in each age group are presented in Table 3.

#### Attention

Analyses were performed to investigate the main effects of age group and sex, and the interaction between age group and sex on the AEFI subscale *attention*. Results revealed a significant main effect of age group, *F* (2, 447) = 3.70, *p* = .03, *η*_*p*_^*2*^ = 0.02. The sex difference approaches significance (*F* (1, 447) = 3.10, *p* = .08, *η*_*p*_^*2*^ < 0.01), and additional investigation of male-female differences in each age group was therefore performed. The interaction between age group and sex was not significant, *F* (2, 447) = 2.10, *p* = .12, *η*_*p*_^*2*^ < 0.01. Results are presented in Table 2.

Additional analyses were performed to investigate differences on the AEFI subscale *attention* between age groups 1 and 2, and between age groups 2 and 3. There was a significant difference between age groups 1 and 2, *F* (1, 292) = 5.14, *p* = .02, *η*_*p*_^*2*^ = 0.02. Mean of age group 1 (*M* = 3.37, *SE* = 0.13) was higher than that of age group 2 (*M* = 2.92, *SE* = 0.14). The difference between age groups 2 and 3 was not statistically significant, *F* (1, 290) = 0.00, *p* > .99, *η*_*p*_^*2*^ = 0.00.

Finally, post-hoc analyses were performed to investigate sex differences on the AEFI subscale *attention* in the three age groups separately. Results revealed a significant sex difference in the second age group, *F* (1,131) = 7.42, *p* < .01, *η*_*p*_^*2*^ = 0.05. Adolescent females (*M* = 3.29, *SE* = 0.19) evaluated attention higher than adolescent males (*M* = 2.52, *SE* = 0.21). The sex differences in the first (*F* (1, 159) = 0.02, *p* = .89, *η*_*p*_^*2*^ = 0.00) and third (*F* (1, 157) = 0.03, *p* = .86, *η*_*p*_^*2*^ = 0.00) age groups were not statistically significant. Mean and standard errors for adolescent males and females in each age group are presented in Table 3.

#### Planning & initiative taking

Analyses were performed to investigate the main effects of age group and sex, and an interaction between age group and sex on the AEFI subscale *planning & initiative taking*. Results revealed that the main effect of age group approaches significance, *F* (2, 447) = 2.84, *p* = .06, *η*_*p*_^*2*^ = 0.01. The main effect of sex (*F* (1, 447) = 1.12, *p* = .29, *η*_*p*_^*2*^ < 0.01) and the interaction between sex and age group were not statistically significant (*F* (2, 447) = 2.15, *p* = .12, *η*_*p*_^*2*^ *=* 0.01). Results are presented in Table 2.

Post-hoc analyses were performed to investigate sex differences on the AEFI subscale *planning & initiative taking* in the three age groups separately. No significant differences between adolescent males and females were found in any of the three age groups (age group 1: *F* (1, 159) = 1.70, *p* = .19, *η*_*p*_^*2*^ = 0.01; age group 2: *F* (1, 131) = 1.36, *p* = .25, *η*_*p*_^*2*^ = 0.01); age group 3: *F* (1, 157) = 2.60, *p* = .11, *η*_*p*_^*2*^ = 0.02). Mean and standard errors for adolescent males and females in each age group are presented in Table 3.

## Discussion

This cross-sectional study examined whether adolescent males and females differ in self-regulation in adolescence. The adolescents evaluated their self-regulation on a self-report questionnaire (the AEFI). Results revealed significant sex differences in self-regulation (total AEFI score) over the three age groups. Adolescent females evaluated their self-regulation higher than adolescent males. Analysis on the level of the three age groups showed that this difference between males and females was confined to middle adolescence. Taking a closer look at this difference on the level of the three AEFI subscales, it was found that this difference was due to the subscales *self-control & self-monitoring* and *attention*, and not to the subscale *planning & initiative taking*.

Females reported higher levels of self-control and self-monitoring, as well as attention than males in middle adolescence. Note that the effect sizes of these differences are small, and the standards errors are equally for adolescent males and females but show that there are substantial variations in the evaluations on a group level. Yet, the sex differences are highly relevant considering the mean difference on the individual level. Note that all items were evaluated on a two-point scale, and mean difference was 0.79 points on the subscale self-control & self-monitoring (SE males is 0.28 and SE females is 0.24) and 0.77 points on the subscale attention (SE males is 0.21 and SE females is 0.19). In view of the average difference per item–which is 0.08 points on the scale *self-control & self-monitoring* (includes 10 items) and 0.12 points on the scale *attention* (includes 6 items)–this is a clear difference between two individuals. This sex-difference is a relevant finding because it shows that the evaluations of 13–16–year-old males and females are congruent with frequently reported differences in behaviour ([63], [64]) and academic performances ([13], [14], [15], [17]) in this age group. The substantially lower levels of self-control and self-monitoring, as well as attention that mid-adolescent males reported in our study may place them at higher risk than females for the engagement in impulsive and anti-social behaviours. There is a substantial literature on this subject, showing that the prevalence of both impulsive behaviour and symptoms on the domain of Attentional Deficits Hyperactive Disorder (i.e., ADHD) in males is much higher than in females (e.g., [65], [66], [67]). With respect to academic performance, the importance of self-regulation is substantiated by our finding that individuals who repeated a grade (and were therefore excluded from our study sample) reported significantly lower levels of self-monitoring and self-control, as well as attention than individuals who did not repeat a grade (see methods for the results). This finding substantiates the idea that the majority of adolescent males may suffer more than the majority of females from the distraction that goes with assessment in a classroom setting. This may negatively affect school performance. The sex differences in self-control and self-monitoring, as well as in attention could thus have far-reaching consequences for both academic achievement and behaviour. For future studies, it would be interesting to explore the relation between the self-perceived self-regulation and the academic achievement and behaviour in each phase of adolescence.

Our findings extend to that of other applied neuropsychological studies which revealed larger sex differences on other cognitive abilities in the period of middle adolescence than in earlier and later adolescence (e.g, sex differences in information processing speed are reported by Camarata and Woodcock [68]; and sex differences in impulse regulation are reported by Cross, Copping & Campbell [69]). In addition to these studies, our findings validate the importance to investigate sex differences in cognitive abilities in adolescence over narrow age classes. In fact, our findings show that if sex differences are investigated in groups with large age ranges (e.g., in 10–to 19–year-olds), the difference in middle adolescence will be unnoticed because of an interaction-effect with age. This is illustrated by the finding on our primary outcome measure–self-regulation–as well as on the subscale attention. Results did not reveal a significant main effect of sex on self-regulation in 13-19-year olds, but we did find a significant difference between mid-adolescent males and females aged 13-15-years. The same occurred on the subscale attention: the main effect of sex in 13-19-year olds approached significance in the expected direction (females rated their attention higher than males), and further examination of sex differences in the mid-adolescent group did reveal a significant main effect of sex. This dilution-effect is caused by the smaller differences in earlier and later adolescence, which equalised average evaluations of males and females over the total age range. As a result, findings are not in line with the large sex differences in behaviour ([63], [64]), cognition ([16], [68]) and academic performance ([17]) that are especially reported in the age period of middle adolescence. This dilution-effect has been shown in one of our earlier studies as well ([70]). In this study, we found sex differences on a mental-rotation task in 7-9-year olds and not in 10-12-year olds. However, this sex difference was not present in the total age-group (i.e., 7-12-year olds). The investigation of sex differences in cognitive abilities in a sample with narrow age-ranges has thus an important advantage compared to earlier studies that examined sex differences in cognitive abilities in age groups with broad age ranges (see for example our earlier study: Lee et al. [20], in which we studied sex differences in 12–17 year olds and see also Huizinga & Smidts, [42] who studied sex differences in 10–18 year olds).

Another advantage of studying sex differences in three separate age groups in adolescence is that it provides insight into the changing magnitude of sex differences in self-regulation over this phase of life. Results of our study suggest that the difference in self-regulation between males and females increases over the course of early adolescence into middle adolescence, to the advantage of females. The difference then starts to decline over the course of later adolescence where males catch up with the females. The present findings–obtained in a cross-sectional study–warrant the execution of a study with a longitudinal design in order to get more insight into the developmental trajectories of self-regulation.

With respect to the question ‘what could be the cause of these male-female differences in self-regulation in adolescence’ it is probable that the sex differences in the trajectory in which the brain matures plays an important role. The brain maturation in middle adolescent males follows another time trajectory compared to females of the same age (see for instance [29], [30], [31]). These male-female differences in brain maturational processes were most pronounced in middle adolescence ([29]). Accordingly, the lack of sex differences in early adolescence as found in the present study could be due to the fact that the differences in brain maturation have not yet been established. Likewise, the sex gap in brain maturation diminishes as males and females grow older. This is also in line with findings of the present study, as no sex differences were observed in the group of late adolescents. Sex differences in brain maturation in middle adolescence may thus contribute to the variations in self-regulation as reported in this study. These differences may underlie the many differences between adolescent males and females on the domains of both behaviour ([63]), cognitive functioning ([17]) and academic performance ([17]). This has quite some implications for applied practice in education.

### Practical implications

The finding that sex differences in self-regulation were only present in middle adolescence is important for educational practice and policy. As we continue to understand learning processes, it is known that not only cognitive abilities are important for learning outcomes. Self-regulation and other neuropsychological abilities, school motivation and attitude towards school are important as well. The adolescent years are a crucial time for developing self-regulatory skills and for putting them into practice. Secondary schooling and working life demand more independence, initiative, and self-reliance. Support of teachers and parents wanes as adolescents get older, because children and teens gain experience and become more independent ([2], [4], [71]). This could be a problem, because the self-regulatory skills of the adolescent are still immature ([1], [2]). Rather than only being content-driven, education should therefore also focus on the learning adolescent in a social setting, and to personal growth. School should implement educational interventions in curricula that stimulate a student’s self-regulatory skills ([72]). This requires an active attitude of educators, who have the important role of giving inspiration and stimulation so that adolescents have the opportunity to develop the skills they need for the future and which support them in anticipating future consequences of present behaviour. Such interventions are promising as they may improve academic achievement and reduce behavioural problems. Our findings imply that especially adolescent males would benefit from these interventions. It is relevant for future research to develop effective interventions and to investigate whether they contribute to better school performance.

### An evaluation of our study

Several aspects of the study need to be addressed to interpret the results correctly. First, in this study self-report measures were used to evaluate the self-regulation of adolescents. Self-reports provide insight in the way individuals perceive themselves and their environment, and in the challenges they encounter. This is important because such a self-evaluation strongly determines how the adolescent lives, how he thinks and how he behaves. Earlier studies on the relation between self-reports of adolescents and behaviour have revealed valuable insights. For instance, Fine, Steinberg, Frick, & Cauffman [73], showed significant relations between self-reported self-control and delinquency in 15-year olds. The fact that mid-adolescent males report more difficulties with the regulation of behaviour and attention than females of the same age could thus have important consequences for behaviour. It is important for future research to elaborate on this possible relation.

A second aspect of the study that needs to be addressed is the use of the AEFI. The AEFI was used to evaluate the self-regulation of adolescents and has been used in earlier large-scale studies in children and adolescents (see for instance [18], [22], [41], [56], [59]). The AEFI as a whole is a compound score and an indicator for self-regulation and related aspects of executive functions ([2]). The instrument allows to evaluate several deeper functions which underlie self-regulation. In our study, the internal consistency of these subscales was re-investigated. The results showed that the internal consistency in the mid-adolescent group on the subscale *planning & initiative taking* is somewhat lower than advised by Clark and Watson ([60]). The finding on this subscale should therefore be interpreted with some caution. It could be that no sex difference exists, and associations observed here reflect a spurious association. But another possibility is that the finding is a more conservative estimation due to low internal consistency. An explanation for the lower correlations could be that items related to “planning ability” as well as to “initiative taking” were grouped together. These two can be considered as separate abilities. The lower correlations highlight the importance to note that the instrument is not used in its psychometric qualities in this study, but as an epidemiological instrument in order to gain information on a group-level and not on an individual level ([74]).

The third aspect to address is the composition of the sample. A strength of our study is the large sample size of the pool of subjects (selected from a large-scale study of Dutch juveniles). It allowed us to control for several age-extrinsic variables by selecting a homogeneous sample from this larger population. It enabled us, first, to exclude those individuals that repeated a class. The decision to exclude these participants was based upon the consideration that the inferior academic performance of these subjects could be due to either issues related to slower neuropsychological development and brain maturation and/or to environmental factors such as poor support and lower SES (see [7] who investigated differences in the neuropsychological development of children with higher and lower educated parents). As a consequence of this procedure, the study sample was homogenised with respect to individual variability in the levels of self-regulation and thereby to variability which could be ascribed to age-extrinsic factors. Second, the study sample was homogenised by including only individuals of a Dutch origin. Participants were asked to indicate their heritage, which is a strength. It provides information on the heritage that the participants themselves believe they belong to, and thus about the norms and values they live by. This is important information because there are many studies which show that the neuropsychological development of an individual is at least partly determined by the cultural background in which they grow up (e.g., [16], [75]). Miller and Halpern [16], for instance, reported that sex differences on cognitive abilities are culture specific. According to these authors, they are present in some cultures, but absent or reversed in others. It is therefore important to note that the findings of the present study cannot directly be generalised towards adolescents from immigrant groups or second-generation ethnic minority groups. Yet, they can be generalised to adolescents growing up industrialised countries with similar developmental pattern of self-regulation (see for instance [52]). By restricting our study to a relatively homogeneous sample consisting of adolescents born from parents who live in the Netherlands for more than one generation in addition to controlling for individual differences in school performance, variance caused by SES has been reduced in our study sample. Nevertheless, some variance could still be present (which could have influenced our results), but this is a methodological issue with all large-scale studies. The lowest SES group is mostly underrepresented as these people are often not in the ability to participate in research. We agree that SES might have influenced the development of self-regulatory abilities, and future investigation of SES with respect to sex differences in each phase of adolescence is worth further examination. It would also be interesting to investigate whether adolescents with another cultural background or heritage report sex differences in their self-regulation, especially given the central role of self-regulation to academic achievement and behavioural problems. Any evidence for sex differences in the various cultures in the period of adolescence may offer important new insights in the underpinnings of adolescents’ daily life decisions, behaviour, social performance and academic achievements. Finally, for establishing the external validity of the present study, it is important for future research to re-investigate our findings in adolescents with equal backgrounds and evaluate self-regulation by objective scales and other measures.

In conclusion, our study revealed differences between adolescent males and females in self-regulation as indicated by self-report. It appears that mid-adolescent males experience more difficulties with self-control and self-monitoring, as well as with attentional functions than females of the same age. This indicates that the development of self-control and self-monitoring, and attentional functions lags behind in males compared to that of females at the age of 13–16 years. This finding is important for educational practice and policy as it could offer explanations for the sex differences that exist in school performances and problem behaviour, especially in middle adolescence. The implication is that mid-adolescent males may benefit from new educational interventions which stimulate the development of self-regulation. These interventions should focus more on improving the awareness to the self-regulation of adolescents. By changing the adolescents’ attitude, interventions may give the adolescent the means to change their behaviour. Educational innovations and dedicated intervention programs could be developed and offered at school, but also in mental health care settings. This approach may bear the promise that sex differences in academic achievement and behavioural problems could be reduced by stimulating the development of self-regulation in adolescent males.

## Supporting information

S1 Table Reliability statistics of the amsterdam executive functioning inventory. (DOCX)
