# Supplementary material for: Sex differences in self-regulation in early, middle and late adolescence: A large-scale cross-sectional study
Source: PLoS One. 2020 Jan 13;15(1):e0227607. doi: 10.1371/journal.pone.0227607 (PMC6957194; doi:10.1371/journal.pone.0227607)
Supplement: S1 Table — (DOCX) [file pone.0227607.s001.docx]

**Supporting information**

**S1 Table. Reliability Statistics of the Amsterdam Executive Functioning Inventory**

|  | **10–12 years old** |  | **13–15 years old** |  | **16–18**  **years old** |  |
| --- | --- | --- | --- | --- | --- | --- |
| **AEFI items** | **Cronbach’s Alpha** | **r_is_** | **Cronbach’s Alpha** | **r_is_** | **Cronbach’s Alpha** | **r_is_** |
| *Attention* | 0.7 |  | 0.7 |  | 0.8 |  |
| 1. I am not able to focus on the same topic for a long period of time |  | 0.5 |  | 0.4 |  | 0.5 |
| 4. I am easily distracted |  | 0.5 |  | 0.6 |  | 0.7 |
| 6. My thoughts easily wander |  | 0.4 |  | 0.5 |  | 0.6 |
|  |  |  |  |  |  |  |
| *Self-control & self-monitoring* | 0.6 |  | 0.6 |  | 0.6 |  |
| 5. I often reacts too fast. I’ve done or said something before it is my turn. |  | 0.4 |  | 0.3 |  | 0.3 |
| 7. It is difficult for me to sit still |  | 0.5 |  | 0.4 |  | 0.4 |
| 10. It takes a lot of effort for me to remember things |  | 0.3 |  | 0.4 |  | 0.4 |
| 11. I often forgets what I have done yesterday |  | 0.3 |  | 0.3 |  | 0.4 |
| 12. I often lose things |  | 0.4 |  | 0.3 |  | 0.3 |
|  |  |  |  |  |  |  |
| *Planning & initiative taking* | 0.6 |  | 0.4 |  | 0.7 |  |
| 2. I can make fast decisions (e.g., in lesson) |  | 0.3 |  | 0.2 |  | 0.5 |
| 3. I am well-organized. For example, I am good at planning what I needs to do during a day |  | 0.3 |  | 0.3 |  | 0.3 |
| 8. It is easy for me to come up with a different solution if I get stuck when solving a problem |  | 0.4 |  | 0.3 |  | 0.5 |
| 9. I am full of new ideas |  | 0.4 |  | 0.3 |  | 0.5 |
| 13. I am curious, I want to know how things work. |  | 0.3 |  | 0.1 |  | 0.3 |

*AEFI = Amsterdam Executive Function Inventory; ris = corrected item–scale correlation.*
